# Development of a deep learning-based MRI diagnostic model for human Brucella spondylitis

**DOI:** 10.1186/s12938-025-01404-6

**Published:** 2025-07-09

**Authors:** Binyang Wang, Jinquan Wei, Zhijun Wang, Pengying Niu, Lvlin Yang, Yanmei Hu, Dan Shao, Wei Zhao

**Affiliations:** 1 Ningxia Institute of Clinical Medicine, The Third Clinical Medicine College, People’s Hospital of Ningxia Hui Autonomous Region, Ningxia Medical University, Zhengyuan Street 301, Yinchuan, 750002 China; 2 College of Computer Science and Technology, Changchun University, Changchun, China; 3 Radiology Department, General Hospital, Ningxia Medical University, Yinchuan, China; 4 Department of Biochemistry and Molecular Biology, Basic Medicine College, Ningxia Medical University, Yinchuan, China

**Keywords:** Brucella spondylitis (BS), Tuberculous spondylitis, Deep learning model, Magnetic resonance image, Spine

## Abstract

**Introduction:**

Brucella spondylitis (BS) and tuberculous spondylitis (TS) are prevalent spinal infections with distinct treatment protocols. Rapid and accurate differentiation between these two conditions is crucial for effective clinical management; however, current imaging and pathogen-based diagnostic methods fall short of fully meeting clinical requirements. This study explores the feasibility of employing deep learning (DL) models based on conventional magnetic resonance imaging (MRI) to differentiate BS and TS.

**Methods:**

A total of 310 subjects were enrolled in our hospital, comprising 209 with BS, 101 with TS. The participants were randomly divided into a training set (*n* = 217) and a test set (*n* = 93). And 74 with other hospital was external validation set. Integrating Convolutional Block Attention Module (CBAM) into the ResNeXt-50 architecture and training the model using sagittal T2-weighted images (T2WI). Classification performance was evaluated using the area under the receiver operating characteristic (AUC) curve, and diagnostic accuracy was compared against general models such as ResNet50, GoogleNet, EfficientNetV2, and VGG16.

**Results:**

The CBAM-ResNeXt model revealed superior performance, with accuracy, precision, recall, F1-score, and AUC from 0.942, 0.940, 0.928, 0.934, 0.953, respectively. These metrics outperformed those of the general models.

**Conclusions:**

The proposed model offers promising potential for the diagnosis of BS and TS using conventional MRI. It could serve as an invaluable tool in clinical practice, providing a reliable reference for distinguishing between these two diseases.

## Introduction

Brucellosis, commonly known as Mediterranean fever, Malta fever, or undulant fever, is a globally endemic zoonotic disease caused by *Brucella spp.* [1], with over 500,000 new cases reported annually and approximately 2.4 billion individuals at risk worldwide [2]. Human Brucellosis is a multisystemic infection, capable of causing widespread organ involvement [3]. Brucella spondylitis (BS), one of the most common manifestations, accounting for 2% to 60% of cases [4]. The infection typically has an incubation period of 2 to 4 weeks, and the clinical course is divided into three stages based on symptom duration: acute (< 3 months), subacute (3–12 months), and chronic (> 12 months) [5]. Early phase symptoms are non-specific, including fever, malaise, excessive sweating, and musculoskeletal pain, particularly in the lower back [6]. Due to the similarity between the clinical manifestations of BS and tuberculous spondylitis (TS) in the acute and subacute stages [7], particularly low back pain, BS is frequently misdiagnosed. Delayed diagnosis and lack of appropriate treatment can lead to severe complications such as spinal deformities and nerve injury, exacerbating both the physical and economic burden on patients [8].

Current laboratory tests for diagnosing BS, including serum agglutination tests and blood cultures, are limited by false positives, prolonged incubation periods, and low sensitivity [9]. Imaging methods such as X-rays, CT and MRI are commonly employed for diagnostic support. While X-rays can reveal vertebral destruction and intervertebral space narrowing, they lack sensitivity in detecting early-stage lesions and cannot provide a definitive diagnosis [10]. CT scans offer earlier detection of vertebral damage and can identify paravertebral abscesses, but suffer from a lack of specificity [11]. MRI, with its multi-sequence and multi-directional capabilities [12], is the most widely used imaging technique in clinical practice. It enables early detection of intervertebral and vertebral body signal alterations, making it valuable for the early diagnosis and differentiation of BS and TS. There are multiple overlapping signs in the imaging manifestations between BS and TS, and to a certain extent, spine orthopedic surgeons rely on clinical experience to differentiate the diagnosis of BS and TS, which is highly subjective and prone to omission and misdiagnosis [13].

Deep learning (DL) models, as computer-aided diagnostic tool, have gained increasing prominence in medical imaging for disease diagnosis, lesion segmentation, and localization tasks [14]. These models leverage deep neural network architectures inspired by the human brain to interpret complex datasets with high efficiency and accuracy [15]. One of the most labor-intensive aspects of radiological interpretation is the identification and classification of regions of interest or lesions, which DL models are particularly well-suited to address [16]. Among them, convolutional Neural Networks (CNNs), a key class of artificial neural networks, have shown powerful performance in image-based applications by automatically learning and extracting hierarchical features using convolutional filters [17]. In the domain of MRI analysis, CNNs have shown remarkable effectiveness, constructing highly discriminative predictive models through multi-level feature extraction from imaging data [18]. DL-based image analysis not only matches the performance of traditional radiological approaches but, in specific clinical scenarios, has been shown to rival or even exceed expert-level diagnostic accuracy. This technological advancement underscores the engineering value of artificial intelligence (AI) in enhancing decision support systems and highlights its broader potential to standardize and elevated the quality of medical imaging diagnostics. In spinal imaging, DL applications have been successfully developed for tasks such as vertebral fractures classification [19], spinal structure recognition [20], intervertebral disc degeneration grading [21], and differentiation of spinal tumors and inflammatory lesions [22]. However, DL models specifically designed to distinguish between BS and TS based on MRI data remain limited. This study proposes to develop a DL model utilizing conventional MRI to accurately differentiate BS from TS. By improving diagnostic precision and enabling earlier clinical intervention, this model holds considerable clinical utility, potentially reducing diagnostic delays, minimizing patient morbidity, and supporting physicians in informed clinical decision-making.

## Results

### General clinical information

A cohort of 310 patients was included in this study, comprising 209 individuals diagnosed with BS and 101 with TS. The cohort was randomly divided into a training set (70%) and a test set (30%). Specifically, the training set included 217 cases (146 BS and 71 TS), while the validation set comprised 93 cases (63 BS and 30 TS). An external validation set of 74 cases (46 BS and 28 TS) was used for model evaluation. A comparative analysis of demographic and clinical characteristics between the BS and TS groups is summarized in Table 1. Statistically significant differences were observed between the two groups in several key variables, including age, gender, and the extent of vertebral involvement. The mean age of patients with BS was 54.69 ± 11.55 years, while those with TS had a mean age of 51.15 ± 19.11 years. Gender distribution revealed a higher proportion of male patients in the BS group (74.16%, n = 155) compared to the TS group (25.48%, *n* = 53). Furthermore, significant differences in the extent of vertebral involvement were observed, as assessed by Chi-squared tests for single, dual and multiple vertebral segment involvement (*P* = 0.003), which was below the threshold for statistical significance (*P* < 0.05). These findings highlight the important epidemiological and clinical distinctions between BS and TS groups, providing a solid foundation for the subsequent model validation and performance assessment.

**Table 1:** Comparison of patient characteristics between BS and TS groups

	Total (n = 310)	BS (n = 209)	TS (n = 101)	*P* value
Age (years)	52.92 ± 15.33	54.69 ± 11.55	51.15 ± 19.11	***P* < **0.001
Gender				
Female	208	155	53	***P* < **0.001
Male	102	54	48	
Single vertebrae	64	33	31	
L1	21	7	14	0.041
L2	3	1	2	0.518
L3	8	3	5	0.395
L4	13	10	3	0.004
L5	19	12	7	0.024
Two vertebrae	211	147	64	
L1 + L2	29	8	21	***P* < **0.001
L2 + L3	27	15	12	0.088
L3 + L4	53	42	11	0.080
L4 + L5	70	56	14	0.021
L5 + S1	32	26	6	0.122
Multiple vertebrae (≥ 3)	35	29	6	

### Comparison of the performance of multiple models

The CBAM-ResNeXt model demonstrated superior diagnostic performance in terms of accuracy, sensitivity, and specificity compared to standard models. A comparison with established models, including GoogleNet, VGG16, and EfficientNetV2, is presented comprehensively in Table 2. The CBAM-ResNeXt model achieved an exceptional accuracy of 0.942, outperforming the ResNet50 model, which achieved 0.876. This high accuracy reflects the model’s ability to minimize missed diagnoses of BS, ensuring fewer cases are overlooked. Both CBAM-ResNeXt and ResNet50 models exhibited the highest specificity values of 0.948 and 0.890, respectively, demonstrating strong performance in accurately identifying non-BS cases. Furthermore, the F1 scores, which balance precision and recall, were significantly higher for CBAM-ResNeXt (0.938) and ResNet50 (0.868), indicating their robust ability to maintain both high precision and recall. Notably, the CBAM-ResNeXt model achieved the highest overall precision (0.939) among the models evaluated. This underscores the model’s consistent performance across multiple metrics, making it the most reliable for clinical diagnosis of BS among those tested. Table 3 presents detailed performance metrics, including accuracy, sensitivity, specificity, and AUC, which clearly illustrate the model’s diagnostic capability on the external dataset.

**Table 2:** Comparison of the diagnostic performance of CBAM-ResNeXt with multiple models

Model	Accuracy	Precision	Recall	F1-score	AUC	Specificity
CBAM-ResNeXt	0.942(95% CI 0.920–0.964)	0.939(95% CI 0.917–0.961)	0.937(95% CI 0.915–0.959)	0.938(95% CI 0.916–0.960)	0.953(95% CI 0.929–0.977)	0.948(95% CI 0.925–0.971)
ResNet50	0.876(95% CI 0.844–0.908)	0.879(95% CI 0.847–0.911)	0.870(95% CI 0.839–0.901)	0.868(95% CI 0.837–0.899)	0.908(95% CI 0.875–0.941)	0.890(95% CI 0.859–0.921)
GoogleNet	0.723(95% CI 0.700–0.764)	0.662(95% CI 0.643–0.681)	0.671(95% CI 0.651–0.691)	0.663(95% CI 0.643–0.683)	0.719(95% CI 0.697–0.741)	0.742(95% CI 0.720–0.764)
VGG16	0.815(95% CI 0.780–0.850)	0.808(95% CI 0.775–0.841)	0.812(95% CI 0.780–0.844)	0.803(95% CI 0.771–0.835)	0.823(95% CI 0.790–0.856)	0.805(95% CI 0.772–0.838)
EfficientNetV2	0.738(95%CI 0.710–0.766)	0.733(95%CI0.707–0.759)	0.729(95% CI0.702–0.756)	0.727(95%CI0.701–0.753)	0.759(95%CI0.738–0.780)	0.728(95%CI0.702–0.754)

**Table 3:** Diagnostic performance of the model on external validation set

Model	Accuracy	Precision	Recall	F1-score	AUC	Specificity
CBAM-ResNeXt	0.934(95% CI 0.913–0.955)	0.932(95% CI 0.911–0.953)	0.930(95% CI 0.908–0.952)	0.931(95% CI 0.910–0.972)	0.948(95% CI 0.924–0.972)	0.942(95% CI 0.919–0.965)
ResNet50	0.872(95% CI 0.843–0.901)	0.882(95% CI 0.854–0.910)	0.865(95% CI 0.837–0.893)	0.873(95% CI 0.845–0.901)	0.903(95% CI 0.876–0.930)	0.895(95% CI 0.868–0.922)
GoogleNet	0.725(95% CI 0.705–0.745)	0.658(95% CI 0.638–0.678)	0.675(95% CI 0.655–0.695)	0.666(95% CI 0.646–0.686)	0.716(95% CI 0.696–0.736)	0.745(95% CI 0.725–0.765)
VGG16	0.820(95% CI 0.790–0.850)	0.812(95% CI 0.782–0.842)	0.805(95% CI 0.775–0.835)	0.808(95% CI 0.778–0.838)	0.815(95% CI 0.785–0.845)	0.810(95% CI 0.780–0.840)
EfficientNetV2	0.741(95% CI 0.716–0.766)	0.728(95%CI 0.705–0.751)	0.735(95% CI 0.712–0.758)	0.722(95%CI 0.699–0.745)	0.763(95%CI 0.745–0.780)	0.733(95%CI 0.709–0.757)

**Fig. 1:**
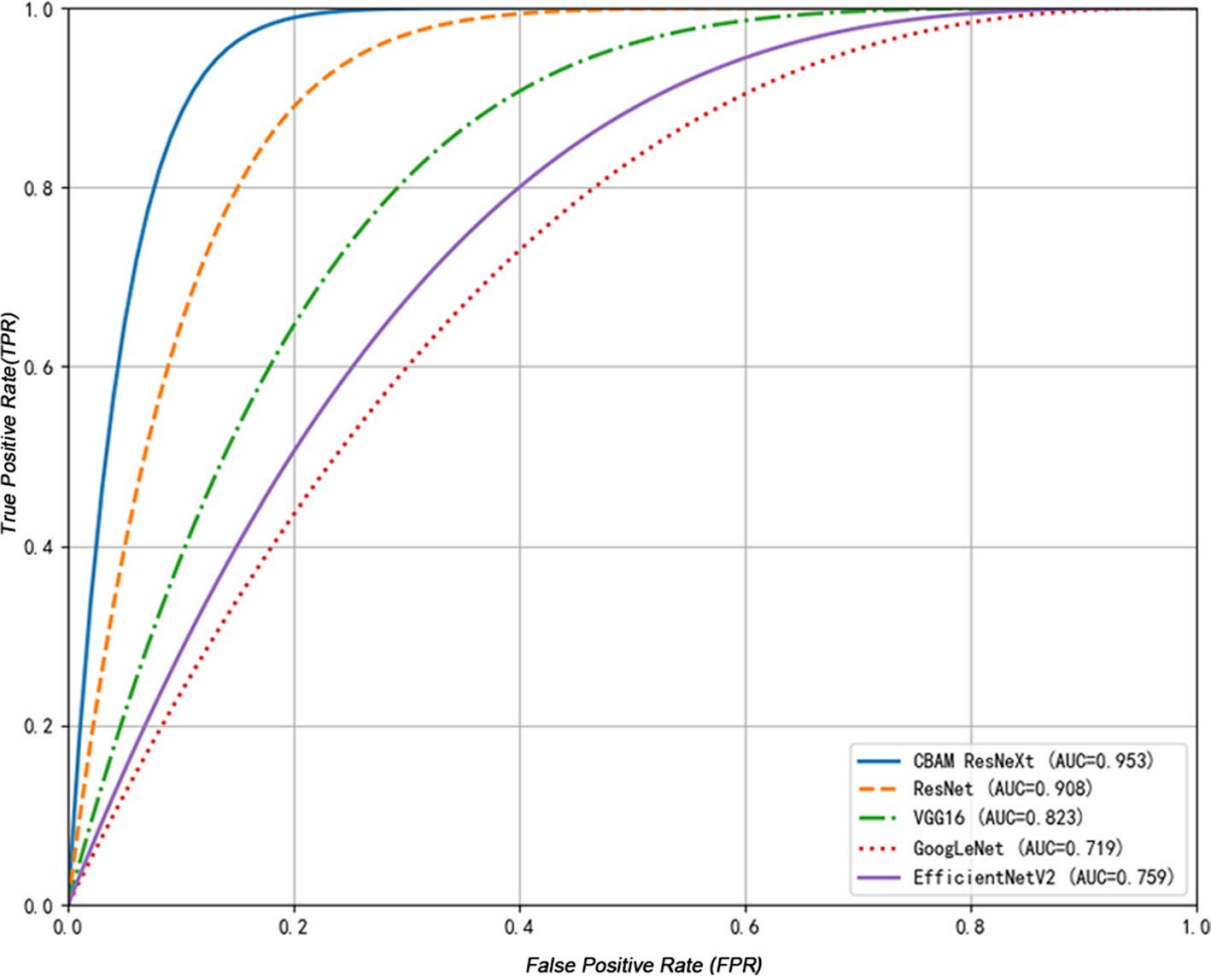
ROC curves for the differential diagnosis models

As depicted in Fig. 1, among all models, the CBAM-ResNeXt model has the highest AUC (0.953).

**Fig. 2:**
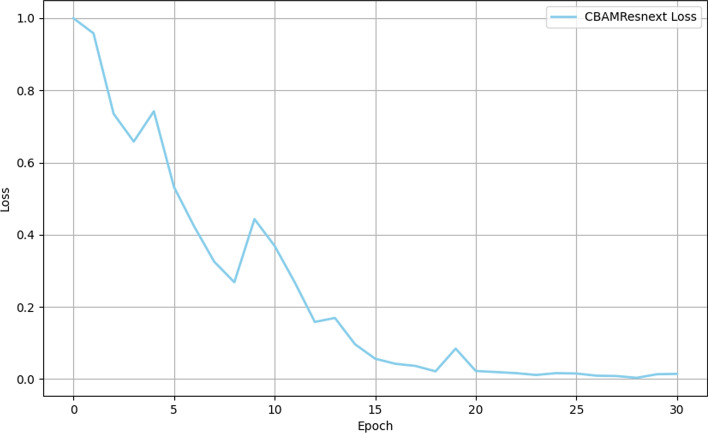
Training loss curve of the CBAM-ResNeXt-50 model

**Fig. 3:**
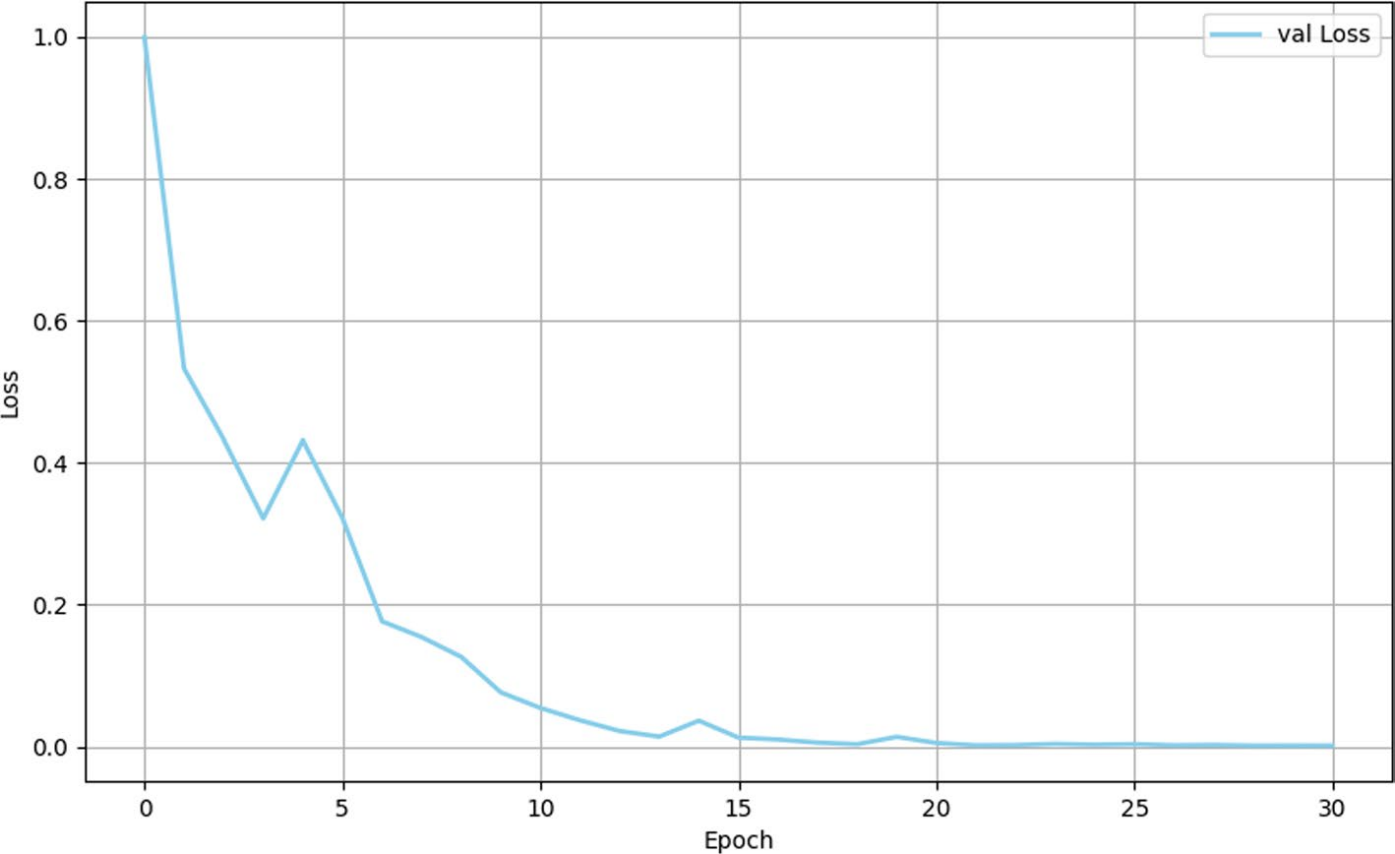
Validation loss curve of the CBAM-ResNeXt-50 model

The model stabilized after approximately 20 iterations, as reflected in the trajectory of the loss function during training (Fig. 2). Initially, a more rapid rate of convergence was observed between iterations 0–15, followed by a gradual stabilization from iterations 15–20. This indicates efficient optimization of model parameters and error minimization throughout the training and test phases (Figs. 2 and 3). Furthermore, a clear inverse relationship between the loss function and accuracy was evident, with a decrease in loss corresponding to an increase in accuracy. This trend aligns with theoretical expectations, where improved accuracy is typically associated with reduced loss. Overall, the model showed robust performance across both training and test datasets, confirming its reliability and effectiveness in diagnostic applications. This was further substantiated by the consistent refinement of model parameters as training progressed.

## Discussion

Brucellosis is a widespread zoonotic infection that can lead to systemic involvement of multiple organs systems, with spinal infection being one of its most frequent complications [23]. Among the osteoarticular manifestations of brucellosis, BS is particularly prevalent due to the spine's complex anatomy and its rich vascular supply [24]. Despite recent advancements, the accurate diagnosis of BS remains challenging, primarily because of its clinical and radiological similarities with TS, leading to frequent diagnostic errors [25]. To address this challenge, we developed and validated a DL model that utilizes conventional spinal MRI T2WI sequences to differentiate BS from TS and to predict the severity of BS-related imaging manifestations. Our findings indicate that this model demonstrates significant promise in the clinical diagnosis of BS. By leveraging nuclear magnetic resonance images, this DL framework not only accurately distinguishes BS from TS, but also shows potential for broader clinical application in diagnosing infectious spine diseases.

BS and TS often present similarly in clinical practice, necessitating confirmatory tests such as blood culture or tissue biopsies. However, these diagnostic methods are time-consuming and can delay timely diagnosis [26]. In this context, spinal imaging, particularly MRI, plays a critical role in the differential diagnosis of these two diseases. Spinal MRI has numerous advantages, including a rapid examination cycle, high resolution, and wide applicability [27]. While BS and TS share similar imaging feature, certain characteristic differences do exist. DL models, as non-invasive diagnostic tools, have shown efficacy in distinguishing between these two conditions [28]. In our study, we evaluated three established DL models alongside our custom-built CBAM-ResNeXt-50 model. Among these, the ResNet-50 model, which incorporates residual blocks with skip connections, stands out for its ability to improve network depth and stability, leading to enhanced accuracy and robustness [29]. The VGG16 model, with 16-layer architecture, is known for its simplicity and depth, which enhances network complexity and is widely utilized for tasks like image classification and segmentation [30]. GoogleNet, with its Inception module, enables multiple convolutional operations within the same layer, improving feature extraction across different scales and increasing model nonlinearity [31].

**Fig. 4:**
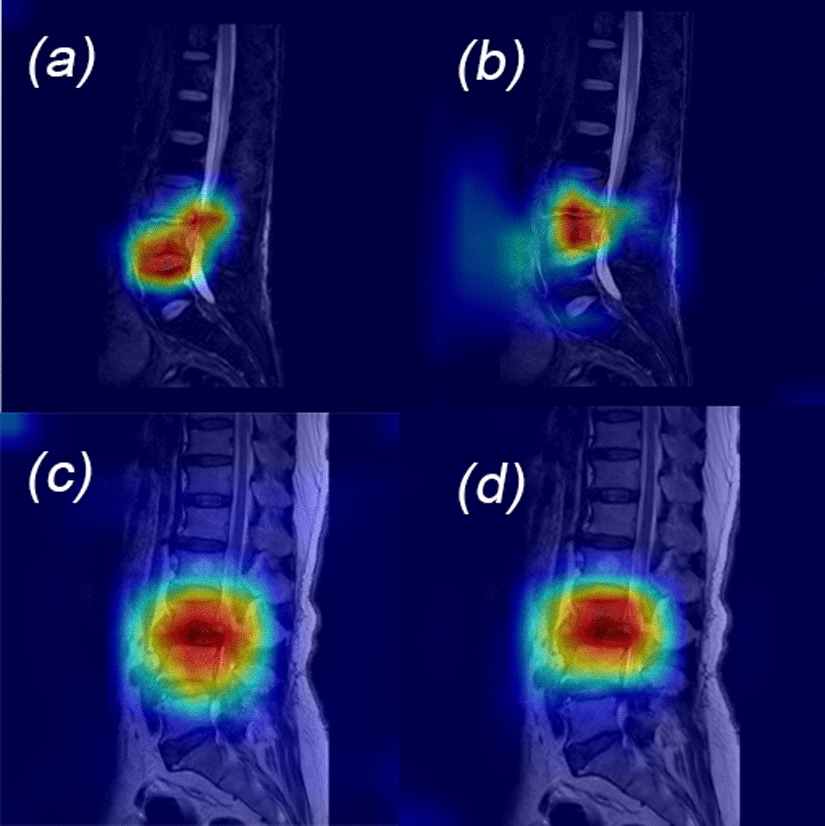
Model identification of lesion area: **a**, **b** for BS, **c**, **d** for TS

To further interpret the features extracted by these DL models, we employed the Gradient-weighted Class Activation Mapping (Grad-CAM). This technique visually highlights the regions of the image that the model focuses on during classification, providing insights into diagnostically relevant areas. As depicted in Fig. 4, the dark red regions identified by Grad-CAM aligned with actual spinal lesions, validating the model's focus on critical diagnostic accurately focusing on lesion regions, which significantly improved diagnostic accuracy for both BS and TS.

Previous studies have also demonstrated that conventional MRI can distinguish between BS and TS qualitatively [12]. Our research further supports this by utilizing T2WI MRI sequences to develop a DL model capable of distinguishing TS from BS, with the T2WI-based model performing exceptionally well. In the broader field of computer-aided diagnosis of spinal diseases, DL models, particularly those based on convolutional neural networks (CNNs), have shown considerable potential for clinical applications. Kim [32] constructed a CNN model to differentiate between TS and pyogenic spondylitis (PS) using MRI images. Their model demonstrated an AUC value of 0.802 for TS vs. PS classification, comparable to the performance of radiologists. Similarly, in the diagnosis of benign vs. malignant spinal fractures, a ResNet50-DL model showed diagnostic accuracy of 92%, with sensitivity and specificity improvements of over 20% compared to clinicians with limited experience [33].

BS typically presents as a mild, non-caseating granulomatous infection with associated bone marrow edema, leading to a relatively uniform increase in signal intensity within the vertebral body. Early-stage BS manifests as lysis on the anterior surface of the superior endplate, where bone destruction and osteophyte proliferation occur simultaneously, leading to sclerosis of the residual bone while preserving vertebral integrity [34]. In contrast, TS typically begins in the anterior portion of the vertebral body adjacent to the endplates, with minimal involvement of the intervertebral discs due to the absence of mycobacterial protein hydrolases [35]. As TS progresses, vertebral destruction intensifies, leading to wedge-shaped deformities, vertebral kyphosis, and narrowing of intervertebral space [36]. TS is frequently characterized by multisegmental involvement, with significant subvertebral or paravertebral spread [37]. While abscesses in BS are rare, they may present as irregularly thickened structures with enhancement and abnormal paravertebral signals [38].

T2WI MRI is particularly effective in detecting myeloedema and can reveal high-intensity vertebral bone signals with nearly intact vertebral heights, which is more indicative of BS than TS [39]. Consequently, T2WI-based DL models are highly effective in identifying BS.

This study has several limitations. First, the sample size was relatively limited, and data were obtained from only two centers. While our external validation demonstrates the model’s potential generalizability, a larger, multi-center cohort would be essential to confirm its broader applicability. Second, the study focused exclusively on two spinal infections (BS and TS), limiting its current diagnostic scope. Future studies incorporating a wider spectrum of spinal pathologies could enhance the model’s discriminative capacity and resistance to diagnostic interference. Lastly, only T2WI sequences were used for model training. Integrating additional imaging modalities, such as T1-weighted or contrast-enhanced sequences, may further improve diagnostic accuracy and the clinical utility of the model.

## Conclusions

The CBAM-ResNeXt-50 model developed in this study offers a promising tool for the accurate differentiation of BS and TS, enhancing diagnostic precision in clinical settings. Given the challenges in distinguishing these two conditions, particularly in resource-limited settings, this DL framework provides significant potential for improving clinical outcomes through faster, more reliable diagnoses. The use of conventional T2WI MRI sequences further emphasized the model’s clinical applicability, particularly in settings where advanced imaging techniques may not be readily available.

## Materials and methods

### Patient population

A total of 310 patients diagnosed with either Brucella spondylitis (BS, *n* = 209) or tuberculous spondylitis (TS, *n* = 101), who were treated at the People's Hospital of Ningxia Hui Autonomous Region from January 2014 to December 2023, were retrospectively collected to form the training and test cohorts. In addition, an external validation set comprising 74 cases (46 BS and 28 TS) was obtained from another independent hospital. The 310 cases from the People's Hospital of Ningxia Hui Autonomous Region were randomly divided into a training set and a test set at a ratio of 7:3. The training set included 217 cases (146 BS, 71 TS), and the test set included 93 patients (63 BS, 30 TS). All 310 patients underwent MRI screening, and diagnostically valuable images were selected for each case. A total of 1388 MRI images were collected from the internal cohort, with 972 images used for training and 416 images for testing. The external validation set consisted of 238 MRI images.

The inclusion criteria specified were a confirmed diagnosis of either BS or TS, supported by lumbar spine MRI documentation. Exclusion criteria encompassed MRI of inadequate quality for analysis, as well as patients with other infectious spinal pathologies or conditions that could compromise the integrity of the MRI images, such as spinal bone tumors or vertebral fractures. The diagnostic criteria for BS were based on the Diagnosis for Brucellosis (National Health Commission of China, WS 269–2019) [40]. Similarly, TS diagnosis was based on one of the following: a positive blood culture; histopathological evidence of tuberculosis, a positive bacterial culture from biopsy tissues, or confirmation via clinical treatment and follow-up.

### Data set establishment

**Fig. 5:**
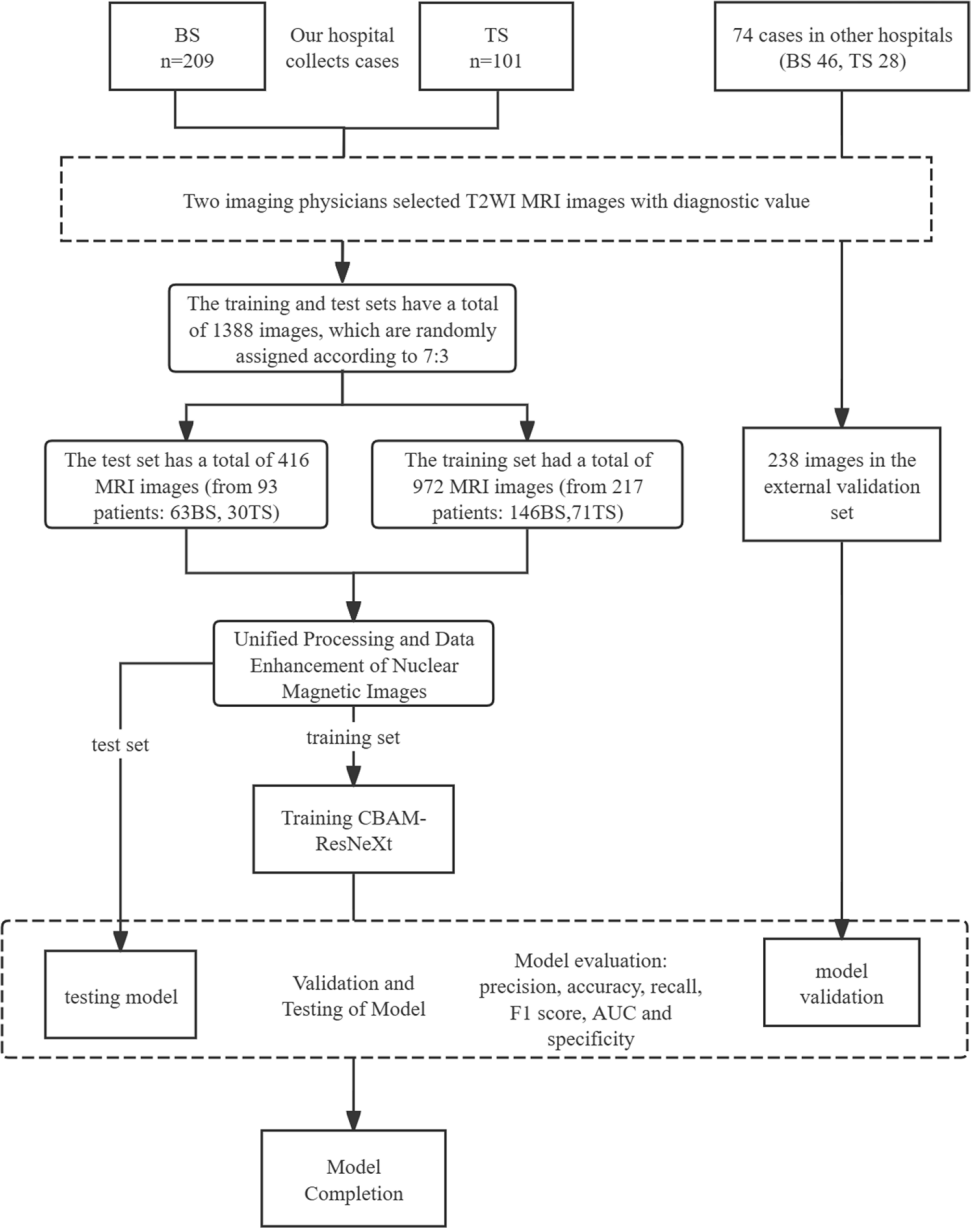
Workflow of the model development and evaluation

MRI images from BS cases (209 individuals) or TS cases (101 individuals) were obtained using a 3.0 Tesla MRI scanner. Specifically, median-sagittal T2WI were exported in JPEG format for subsequent analysis. Image selection was conducted by two independent radiologists for reviewing the MRI images. Two imaging doctors independently selected all the patients' MRI images, and the selection criterion was 3–5 MRI images with diagnostic significance (T2WI MRI images with abnormal results, such as vertebral body and intervertebral space with high signal manifestation), because in a group of MRI images, there are only 3–5 images with diagnostic value, and the others are not very significant for the diagnosis, so there are 3–5 MRI images of each patient used as the model's training, testing and validation. The results were summarized between the two doctors, and when different opinions were encountered, the results were obtained after discussion between the two doctors and the same opinion. Only images that were selected by both radiologists were included in the final dataset. The dataset was then divided into a training set and a test set. As depicted in Fig. 5, the training set, consisting of 972 images, representing approximately 70% of the total dataset, while the test set comprised 416 images, accounting for the remaining 30%. This stratified division was designed to optimize model training and ensure robust evaluation of diagnostic performance. The external validation set consisted of 238 MRI images.

In our hospital, the image acquisition equipment for the MRI data collected in our hospital included: a Philips Ingenia CS3.0 T, 16CH channel PHC posterior Coil, 3.0 T full spinal posterior coil, with the following imaging parameters: T2WI sequence, echo time = 80 ms, repetition time = 2861 ms, field of view = 200 × 280 mm, flip angle = 90°, slice thickness = 5 mm. The external test set was from another hospital with the same inclusion and exclusion criteria as in our hospital. The external hospital data were obtained using a GE SIGNA Architect 3.0 T Scanner, Spine posterior 40CH Coil, 3.0 T full spine posterior coil, the imaging parameters were as follows: T2WI sequence, echo time = 90 ms, repetition time = 2639 ms, field of view = 200 × 320 mm, flip angle = 90°, slice thickness = 4 mm.

### Image data preprocessing: quality control and resizing

For optimal model performance, the MRI images underwent a series of preprocessing steps. The original T2-weighted axial image had a spatial resolution of 512 × 512 pixels, with a horizontal and vertical DPI of 72 and a bit depth of 24 bits. During preprocessing, all images were resized to 512 × 512 pixels to standardize the input dimensions for the model. This resizing was performed to ensure consistency in spatial scale across all images while preserving key structural features essential for analysis. Regarding the average pixel intensity setting of 45, this value was determined through systematic experimentation rather than arbitrary selection. We conducted multiple rounds of tests across a luminance range of 20 to 60, evaluating structural clarity, lesion risibility, and model performance at each level. Our results showed that an average luminance of 45 yielded the most distinct vertebral boundaries, optimal contrast of structural details, and clearer lesion textures. Additionally, under this setting, model feature extraction was most effective, and the validation accuracy improved by approximated 2.7% compared to luminance values of 35 and 55. Furthermore, the training process exhibited greater stability, with a smoother loss curve. Importantly, during brightness adjustment, we preserved the original grayscale dynamic range and bit depth of the images. Only linear intensity normalization was applied, ensuring that overall image quality remained unaffected.

To enhance the model's ability to distinguish key pathological features, we adopted a standardized brightness value during preprocessing. This adjustment was carefully selected to emphasize diagnostically relevant regions while preserving the original grayscale dynamic range and bit depth. Specifically, we applied linear intensity normalization without altering the intrinsic image properties, thereby minimizing the risk of degradation in image quality. To further improve model generalizability and reduce the risk of overfitting, we employed a comprehensive data augmentation strategy. This included random horizontal and vertical flipping—commonly used in medical imaging analysis to artificially expand dataset variability. These augmentation techniques were designed to expose the model to a broader range of anatomical presentations and imaging variations, thereby enhancing its robustness and ability to generalize to previously unseen data.

### Model architecture

**Fig. 6:**
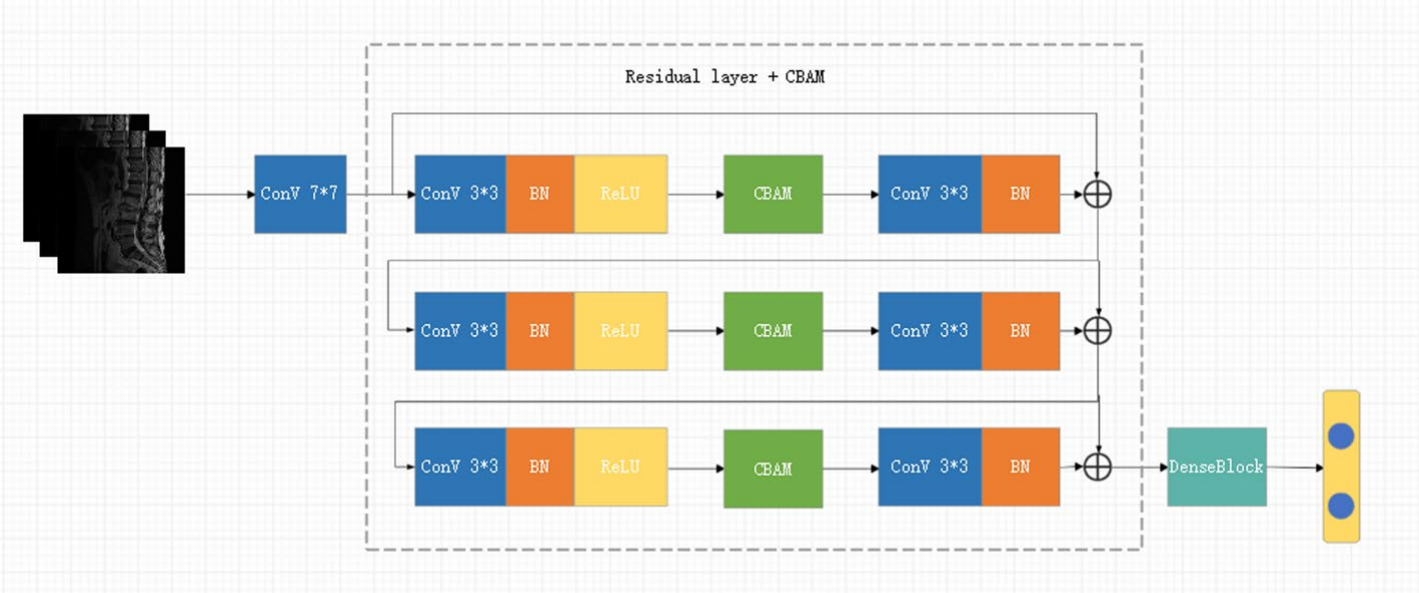
Schematic diagram of the CBAM-ResNeXt-50 model

**Fig. 7:**
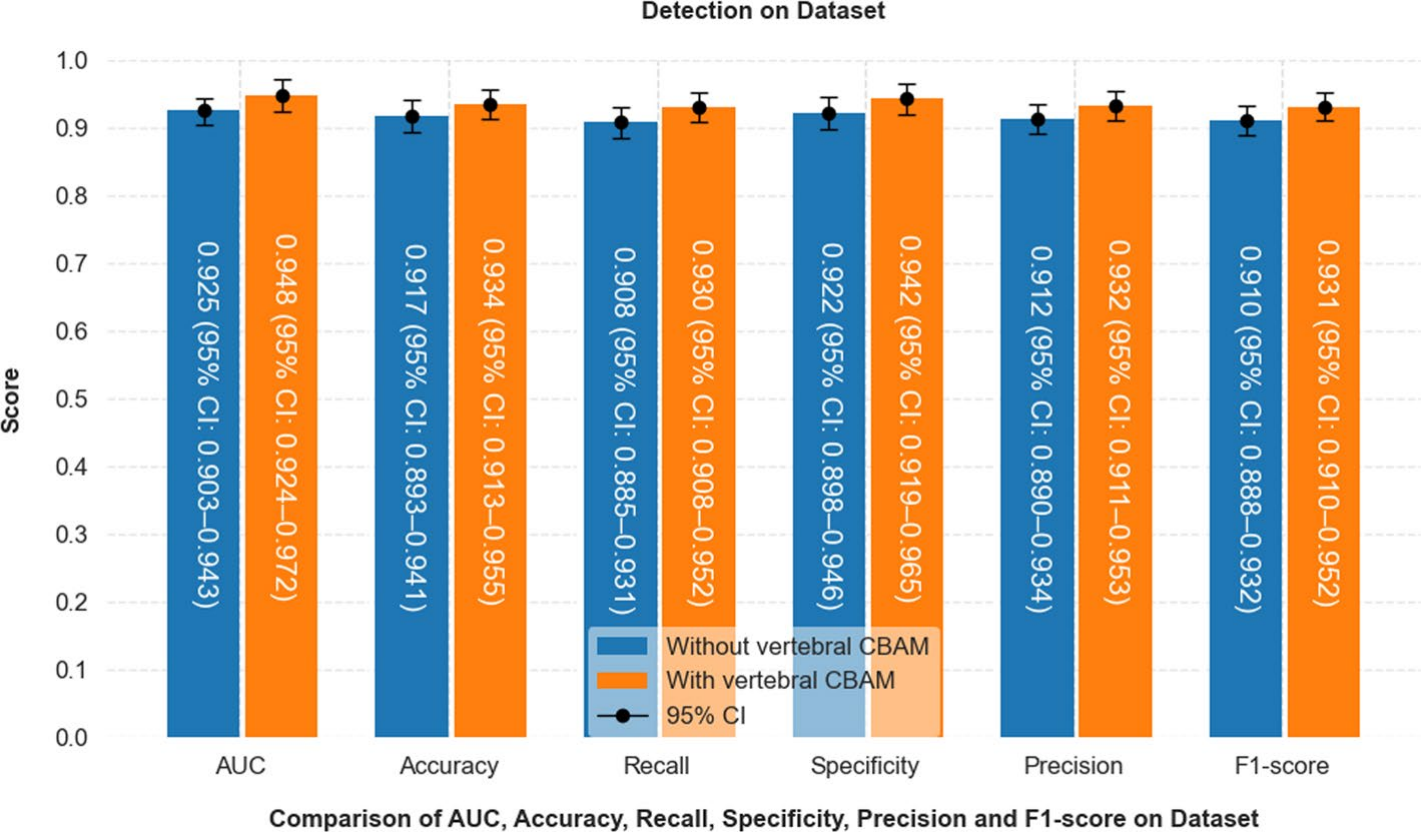
Diagnostic performance of the ResNeXt-50 model with and without the CBAM model

As depicted in Fig. 6, the integration of the CBAM [41] into the ResNeXt-50 architecture is designed to enhance feature representation and improve performance in complex image classification tasks. We chose ResNeXt-50 as the base network considering its enhanced flexibility and expressiveness of feature extraction by introducing grouped convolution (Cardinality), which is particularly suitable for processing medical images with complex structures and subtle details. In this study, Mycobacterium brucei spondylitis BS and TS have highly similar image representations in MR images, such as blurred vertebral edges and overlapping ranges of signal alteration, which make it difficult to achieve effective differentiation by conventional feature extraction alone. To enhance the model's ability to perceive critical regions, we introduced the CBAM module based on ResNeXt-50. CBAM can adaptively guide the model to focus on discriminative regions and features during the training process by means of the series-connected channels and the spatial attention mechanism, thus improving the classification accuracy. We compare the classification effects when the CBAM module is introduced with and without the CBAM module, and the experimental results show that the model with integrated CBAM improves the classification accuracy on the validation set by about 3.1% on average, and the ability to focus on the lesion region is significantly enhanced, which verifies the effectiveness of the module in this task (in Fig. 7). The ResNeXt-50 model serves as the backbone of our approach, consisting of 16 residual blocks and a fully connected layer, structured to efficiently capture intricate patterns within the dataset. The image processing pipeline begins with an initial convolutional layer, which expands the feature dimension to 64 using a 7 × 7 convolutional kernel with a stride of 2. This is followed by a max-pooling layer to reduce spatial dimensions. The data then pass through four stages of residual blocks (also referred to as Conv2 through Conv5 in the standard ResNeXt-50 architecture), each composed of multiple residual blocks and integrated with CBAM modules. Specifically, the residual block configuration follows the standard ResNeXt-50 design: three residual blocks in Stage 1 (Conv2), four in Stage 2 (Conv3), six in Stage 3 (Conv4), and three in the final Stage 4 (Conv5). Each residual block is equipped with 32 parallel convolutional paths, which refers to the grouped convolution strategy employed in the ResNeXt architecture, rather than 32 entirely independent convolutional branches. In this configuration, the input feature maps are split into 32 groups, and each group is processed by a separate 3 × 3 convolutional kernel. This design allows for increased model capacity and representational power without a significant increase in computational cost, as the grouped structure reduces parameter redundancy. The outputs from the 32 groups are then concatenated and passed through the subsequent layers of the residual block.

**Fig. 8:**
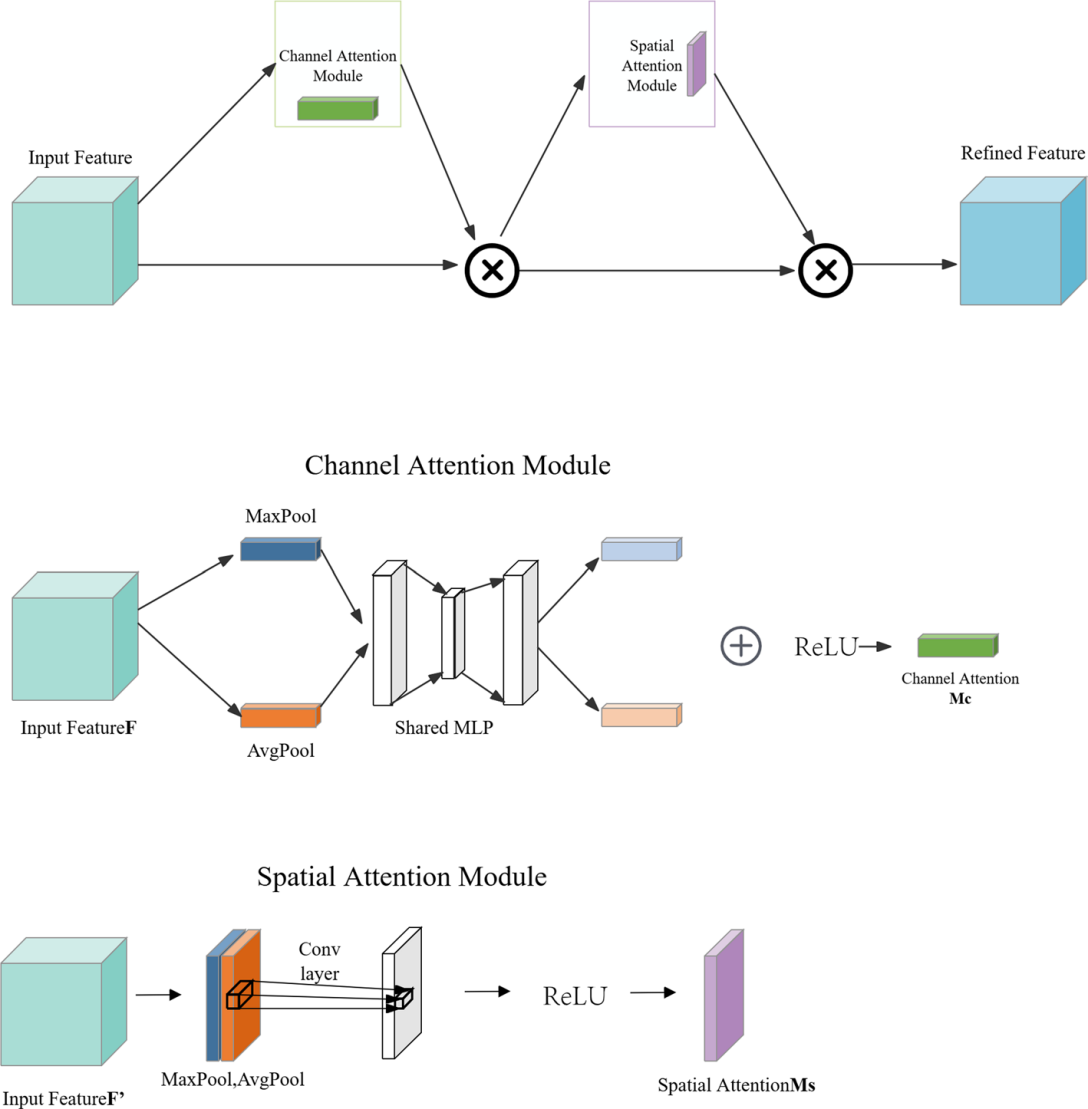
Schematic diagram of the CBAM model

The CBAM modules are strategically integrated after each residual block to recalibrate the feature maps using a dual-attention mechanism. The first component, a channel attention mechanism, focuses on selecting relevant feature channels, while the second component, spatial attention, highlights important spatial regions within the feature maps. This dual-attention mechanism allows the model to focus on the most informative areas, improving both interpretability and performance in Fig. 8. Following the attention recalibration, the data flow through residual connections, which help preserve essential features and ensure that important information is retained throughout the learning process. The feature maps produced by the residual blocks are passed sequentially from one stage to the next. That is, the output of each residual stage (e.g., Conv2) is used as the input for the next stage (e.g., Conv3), without concatenation or averaging between stages. After the final residual block (Stage 4/Conv5), the resulting feature map is fed into a global average pooling (GAP) layer, which reduces the spatial dimensions to a single vector by computing the average value of each channel. This pooled feature vector is then passed into a fully connected (Dense) layer to generate the final classification output.

By combining the ResNeXt architecture with CBAM, our model achieves robust performance in differentiating between BS and TS, demonstrating enhanced accuracy and reliability in medical image classification tasks.

All experiments were conducted using Python 3.8 and the PyTorch deep learning framework (version 1.11.0). The code was executed on a workstation running Ubuntu 20.04, equipped with CUDA 11.3, an NVIDIA RTX 3090 GPU (24 GB memory), and a 14-core Intel^®^ Xeon^®^ Gold 6330 CPU at 2.00 GHz. For model training, the Adam optimizer was employed with a batch size of 32 and an initial learning rate of 0.001. The network was trained for 30 epochs. Prior to training, all MR images were resized to 512 × 512 pixels as part of the preprocessing pipeline to ensure uniform input dimensions across the dataset.

### Statistical analysis and model performance evaluation

Statistical analyses were performed to assess differences between continuous and categorical variables using independent two-sample *t*-tests and Chi-squared tests, respectively. To evaluate the performance of various AI models in diagnosing BS, we used an internal test dataset comprising 310 samples. Key performance metrics, including accuracy, sensitivity, specificity, and the area under the receiver operating characteristic (ROC) curve (AUC), along with F1 scores, were calculated to quantify model efficacy. Model performance was further visualized through ROC curves, with the AUC serving as a quantitative measure of overall diagnostic capability. A threshold of *P* < 0.05 was considered statistically significant for all comparisons. All statistical computations were performed using Python version 3.8, with the sklearn library facilitating model evaluation and analysis. This rigorous statistical approach ensures the robustness and reliability of the results, enabling a comprehensive assessment of AI model performance in medical diagnostics.

## Data Availability

No datasets were generated or analysed during the current study.
